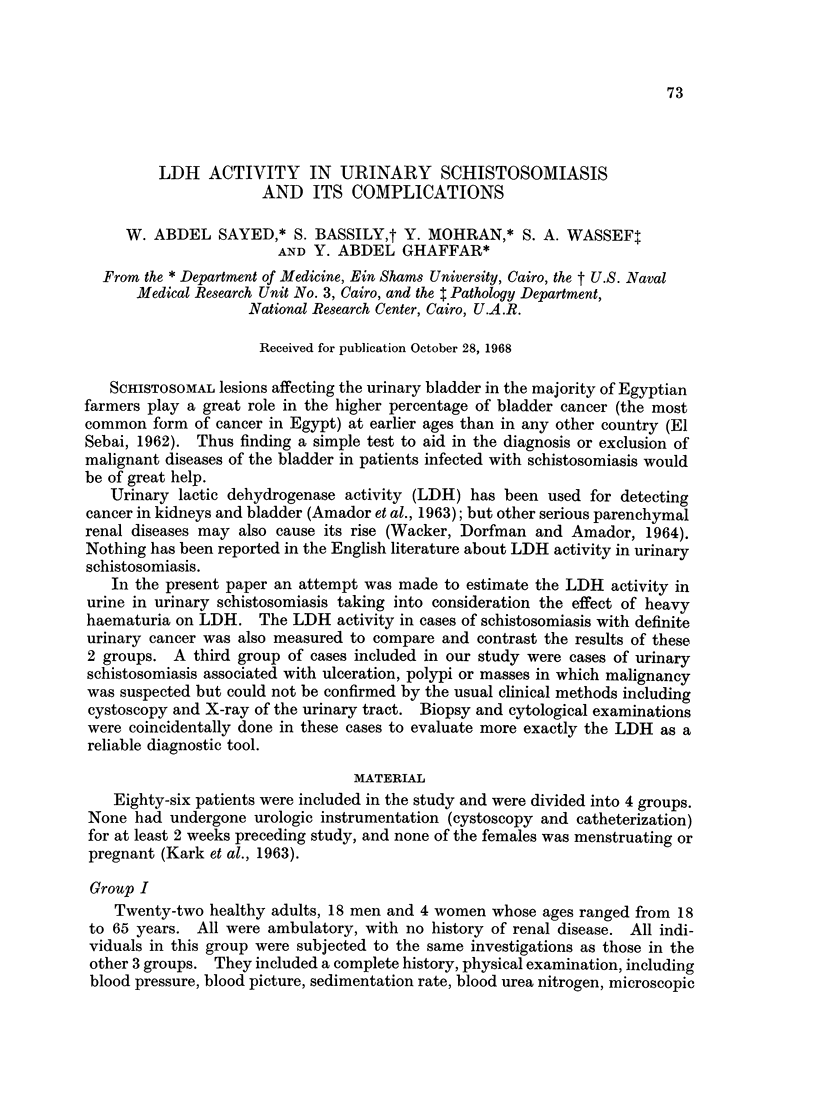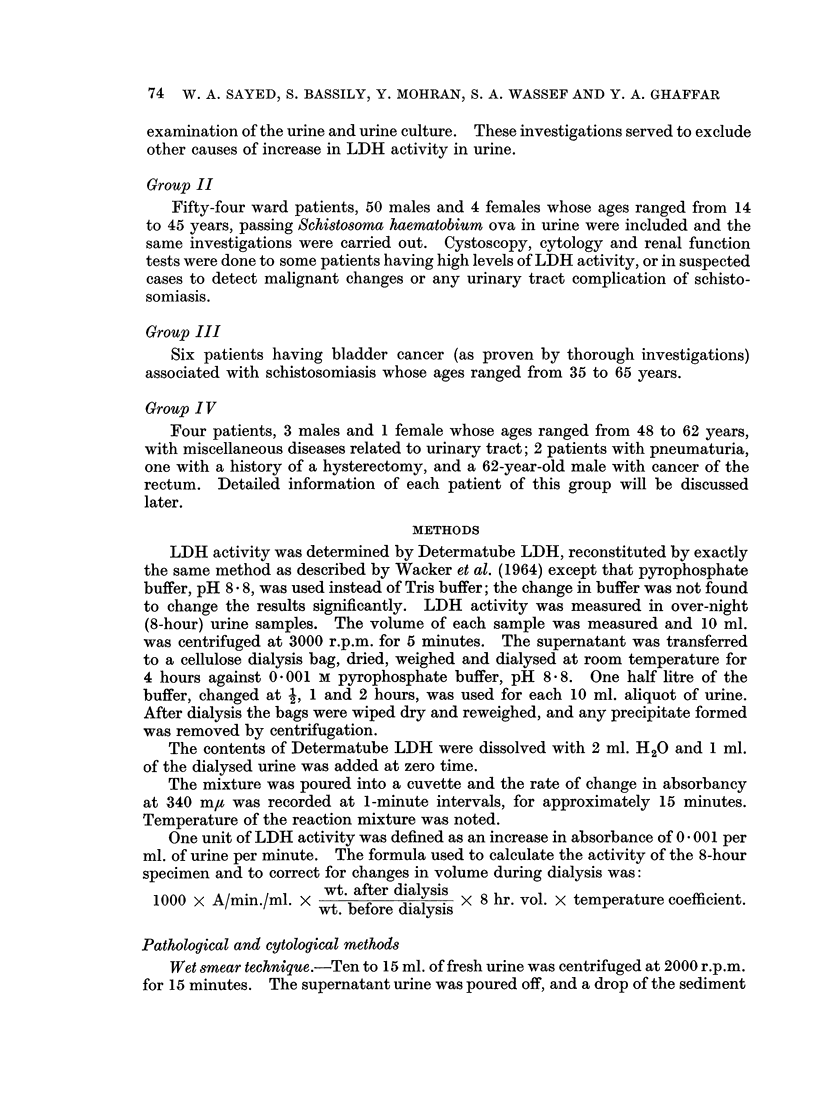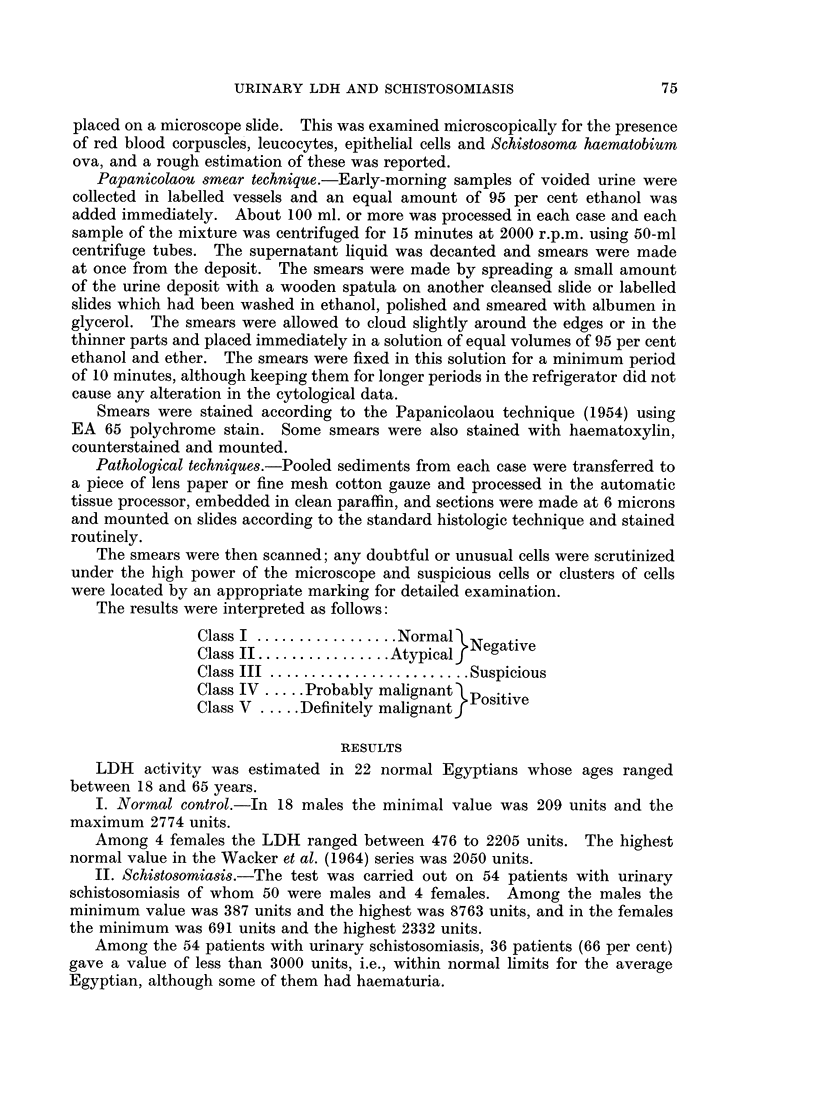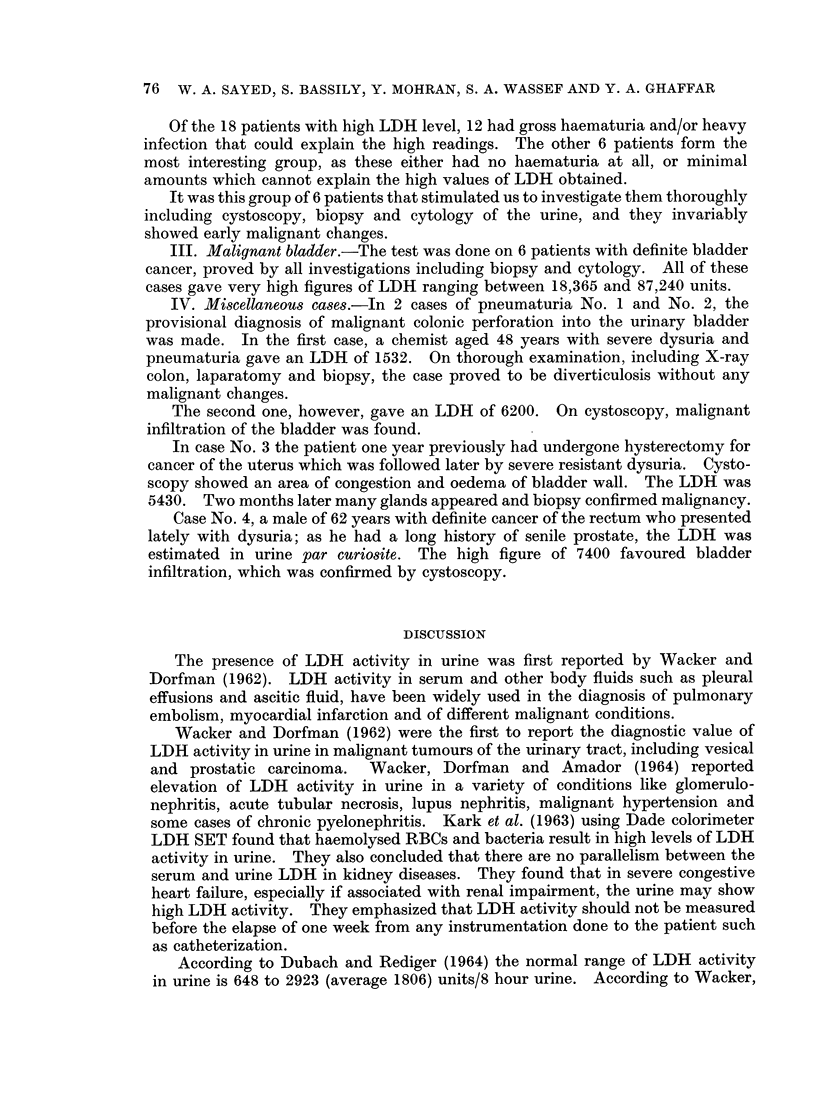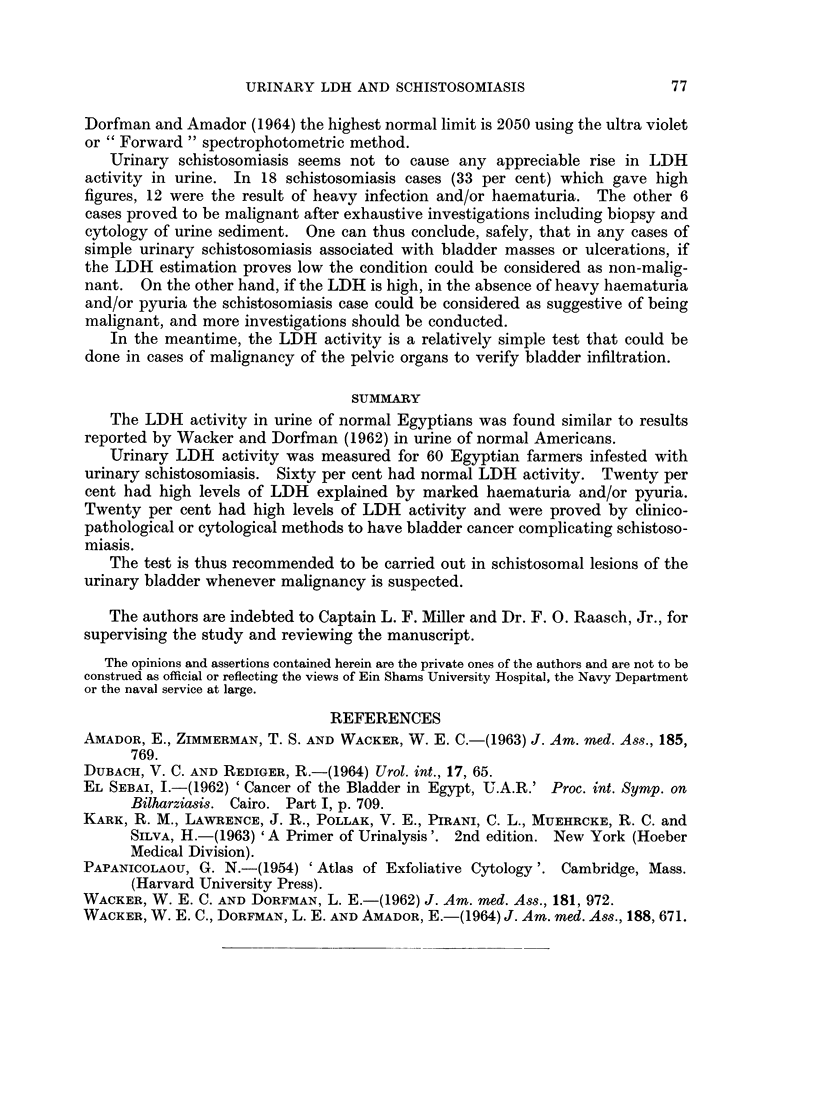# LDH activity in urinary schistosomiasis and its complications.

**DOI:** 10.1038/bjc.1969.12

**Published:** 1969-03

**Authors:** W. A. Sayed, S. Bassily, Y. Mohran, S. A. Wassef, Y. A. Ghaffar


### LDH ACTIVITY IN URINARY SCHISTOSOMIASIS AND ITS COMPLICATIONS

W. ABDEL SAYED,* S. BASSILY,† Y. MOHRAN,* S. A. WASSEF‡ AND Y. ABDEL GHAFFAR*

*From the * Department of Medicine, Ein Shams University, Cairo, the † U.S. Naval Medical Research Unit No. 3, Cairo, and the ‡ Pathology Department, National Research Center, Cairo, U.A.R.*

Received for publication October 28, 1968

SCHISTOSOMAL lesions affecting the urinary bladder in the majority of Egyptian farmers play a great role in the higher percentage of bladder cancer (the most common form of cancer in Egypt) at earlier ages than in any other country (El Sebai, 1962). Thus finding a simple test to aid in the diagnosis or exclusion of malignant diseases of the bladder in patients infected with schistosomiasis would be of great help.

Urinary lactic dehydrogenase activity (LDH) has been used for detecting cancer in kidneys and bladder (Amador *et al.*, 1963); but other serious parenchymal renal diseases may also cause its rise (Wacker, Dorfman and Amador, 1964). Nothing has been reported in the English literature about LDH activity in urinary schistosomiasis.

In the present paper an attempt was made to estimate the LDH activity in urine in urinary schistosomiasis taking into consideration the effect of heavy haematuria on LDH. The LDH activity in cases of schistosomiasis with definite urinary cancer was also measured to compare and contrast the results of these 2 groups. A third group of cases included in our study were cases of urinary schistosomiasis associated with ulceration, polypi or masses in which malignancy was suspected but could not be confirmed by the usual clinical methods including cystoscopy and X-ray of the urinary tract. Biopsy and cytological examinations were coincidentally done in these cases to evaluate more exactly the LDH as a reliable diagnostic tool.

### MATERIAL

Eighty-six patients were included in the study and were divided into 4 groups. None had undergone urologic instrumentation (cystoscopy and catheterization) for at least 2 weeks preceding study, and none of the females was menstruating or pregnant (Kark *et al.*, 1963).

### *Group I*

Twenty-two healthy adults, 18 men and 4 women whose ages ranged from 18 to 65 years. All were ambulatory, with no history of renal disease. All individuals in this group were subjected to the same investigations as those in the other 3 groups. They included a complete history, physical examination, including blood pressure, blood picture, sedimentation rate, blood urea nitrogen, microscopic examination of the urine and urine culture. These investigations served to exclude other causes of increase in LDH activity in urine.

*Group II*

Fifty-four ward patients, 50 males and 4 females whose ages ranged from 14 to 45 years, passing *Schistosoma haematobium* ova in urine were included and the same investigations were carried out. Cystoscopy, cytology and renal function tests were done to some patients having high levels of LDH activity, or in suspected cases to detect malignant changes or any urinary tract complication of schistosomiasis.

*Group III*

Six patients having bladder cancer (as proven by thorough investigations) associated with schistosomiasis whose ages ranged from 35 to 65 years.

*Group IV*

Four patients, 3 males and 1 female whose ages ranged from 48 to 62 years, with miscellaneous diseases related to urinary tract; 2 patients with pneumaturia, one with a history of a hysterectomy, and a 62-year-old male with cancer of the rectum. Detailed information of each patient of this group will be discussed later.

### METHODS

LDH activity was determined by Determatube LDH, reconstituted by exactly the same method as described by Wacker *et al.* (1964) except that pyrophosphate buffer, pH 8·8, was used instead of Tris buffer; the change in buffer was not found to change the results significantly. LDH activity was measured in over-night (8-hour) urine samples. The volume of each sample was measured and 10 ml. was centrifuged at 3000 r.p.m. for 5 minutes. The supernatant was transferred to a cellulose dialysis bag, dried, weighed and dialysed at room temperature for 4 hours against 0·001 M pyrophosphate buffer, pH 8·8. One half litre of the buffer, changed at $\frac{1}{2}$, 1 and 2 hours, was used for each 10 ml. aliquot of urine. After dialysis the bags were wiped dry and reweighed, and any precipitate formed was removed by centrifugation.

The contents of Determatube LDH were dissolved with 2 ml. $H_2O$ and 1 ml. of the dialysed urine was added at zero time.

The mixture was poured into a cuvette and the rate of change in absorbancy at 340 m$\mu$ was recorded at 1-minute intervals, for approximately 15 minutes. Temperature of the reaction mixture was noted.

One unit of LDH activity was defined as an increase in absorbance of 0·001 per ml. of urine per minute. The formula used to calculate the activity of the 8-hour specimen and to correct for changes in volume during dialysis was:

$$1000 \times \text{A/min./ml.} \times \frac{\text{wt. after dialysis}}{\text{wt. before dialysis}} \times \text{8 hr. vol.} \times \text{temperature coefficient.}$$

*Pathological and cytological methods*

*Wet smear technique.*—Ten to 15 ml. of fresh urine was centrifuged at 2000 r.p.m. for 15 minutes. The supernatant urine was poured off, and a drop of the sediment placed on a microscope slide. This was examined microscopically for the presence of red blood corpuscles, leucocytes, epithelial cells and *Schistosoma haematobium* ova, and a rough estimation of these was reported.

*Papanicolaou smear technique.*—Early-morning samples of voided urine were collected in labelled vessels and an equal amount of 95 per cent ethanol was added immediately. About 100 ml. or more was processed in each case and each sample of the mixture was centrifuged for 15 minutes at 2000 r.p.m. using 50-ml centrifuge tubes. The supernatant liquid was decanted and smears were made at once from the deposit. The smears were made by spreading a small amount of the urine deposit with a wooden spatula on another cleansed slide or labelled slides which had been washed in ethanol, polished and smeared with albumen in glycerol. The smears were allowed to cloud slightly around the edges or in the thinner parts and placed immediately in a solution of equal volumes of 95 per cent ethanol and ether. The smears were fixed in this solution for a minimum period of 10 minutes, although keeping them for longer periods in the refrigerator did not cause any alteration in the cytological data.

Smears were stained according to the Papanicolaou technique (1954) using EA 65 polychrome stain. Some smears were also stained with haematoxylin, counterstained and mounted.

*Pathological techniques.*—Pooled sediments from each case were transferred to a piece of lens paper or fine mesh cotton gauze and processed in the automatic tissue processor, embedded in clean paraffin, and sections were made at 6 microns and mounted on slides according to the standard histologic technique and stained routinely.

The smears were then scanned; any doubtful or unusual cells were scrutinized under the high power of the microscope and suspicious cells or clusters of cells were located by an appropriate marking for detailed examination.

The results were interpreted as follows:

| | | |
|---|---|---|
| Class I | Normal | Negative |
| Class II | Atypical | |
| Class III | | Suspicious |
| Class IV | Probably malignant | Positive |
| Class V | Definitely malignant | |

### RESULTS

LDH activity was estimated in 22 normal Egyptians whose ages ranged between 18 and 65 years.

I. *Normal control.*—In 18 males the minimal value was 209 units and the maximum 2774 units.

Among 4 females the LDH ranged between 476 to 2205 units. The highest normal value in the Wacker *et al.* (1964) series was 2050 units.

II. *Schistosomiasis.*—The test was carried out on 54 patients with urinary schistosomiasis of whom 50 were males and 4 females. Among the males the minimum value was 387 units and the highest was 8763 units, and in the females the minimum was 691 units and the highest 2332 units.

Among the 54 patients with urinary schistosomiasis, 36 patients (66 per cent) gave a value of less than 3000 units, i.e., within normal limits for the average Egyptian, although some of them had haematuria.

Of the 18 patients with high LDH level, 12 had gross haematuria and/or heavy infection that could explain the high readings. The other 6 patients form the most interesting group, as these either had no haematuria at all, or minimal amounts which cannot explain the high values of LDH obtained.

It was this group of 6 patients that stimulated us to investigate them thoroughly including cystoscopy, biopsy and cytology of the urine, and they invariably showed early malignant changes.

III. *Malignant bladder*.—The test was done on 6 patients with definite bladder cancer, proved by all investigations including biopsy and cytology. All of these cases gave very high figures of LDH ranging between 18,365 and 87,240 units.

IV. *Miscellaneous cases*.—In 2 cases of pneumaturia No. 1 and No. 2, the provisional diagnosis of malignant colonic perforation into the urinary bladder was made. In the first case, a chemist aged 48 years with severe dysuria and pneumaturia gave an LDH of 1532. On thorough examination, including X-ray colon, laparatomy and biopsy, the case proved to be diverticulosis without any malignant changes.

The second one, however, gave an LDH of 6200. On cystoscopy, malignant infiltration of the bladder was found.

In case No. 3 the patient one year previously had undergone hysterectomy for cancer of the uterus which was followed later by severe resistant dysuria. Cystoscopy showed an area of congestion and oedema of bladder wall. The LDH was 5430. Two months later many glands appeared and biopsy confirmed malignancy.

Case No. 4, a male of 62 years with definite cancer of the rectum who presented lately with dysuria; as he had a long history of senile prostate, the LDH was estimated in urine *par curiosite*. The high figure of 7400 favoured bladder infiltration, which was confirmed by cystoscopy.

### DISCUSSION

The presence of LDH activity in urine was first reported by Wacker and Dorfman (1962). LDH activity in serum and other body fluids such as pleural effusions and ascitic fluid, have been widely used in the diagnosis of pulmonary embolism, myocardial infarction and of different malignant conditions.

Wacker and Dorfman (1962) were the first to report the diagnostic value of LDH activity in urine in malignant tumours of the urinary tract, including vesical and prostatic carcinoma. Wacker, Dorfman and Amador (1964) reported elevation of LDH activity in urine in a variety of conditions like glomerulonephritis, acute tubular necrosis, lupus nephritis, malignant hypertension and some cases of chronic pyelonephritis. Kark *et al.* (1963) using Dade colorimeter LDH SET found that haemolysed RBCs and bacteria result in high levels of LDH activity in urine. They also concluded that there are no parallelism between the serum and urine LDH in kidney diseases. They found that in severe congestive heart failure, especially if associated with renal impairment, the urine may show high LDH activity. They emphasized that LDH activity should not be measured before the elapse of one week from any instrumentation done to the patient such as catheterization.

According to Dubach and Rediger (1964) the normal range of LDH activity in urine is 648 to 2923 (average 1806) units/8 hour urine. According to Wacker,

Dorfman and Amador (1964) the highest normal limit is 2050 using the ultra violet or " Forward " spectrophotometric method.

Urinary schistosomiasis seems not to cause any appreciable rise in LDH activity in urine. In 18 schistosomiasis cases (33 per cent) which gave high figures, 12 were the result of heavy infection and/or haematuria. The other 6 cases proved to be malignant after exhaustive investigations including biopsy and cytology of urine sediment. One can thus conclude, safely, that in any cases of simple urinary schistosomiasis associated with bladder masses or ulcerations, if the LDH estimation proves low the condition could be considered as non-malignant. On the other hand, if the LDH is high, in the absence of heavy haematuria and/or pyuria the schistosomiasis case could be considered as suggestive of being malignant, and more investigations should be conducted.

In the meantime, the LDH activity is a relatively simple test that could be done in cases of malignancy of the pelvic organs to verify bladder infiltration.

### SUMMARY

The LDH activity in urine of normal Egyptians was found similar to results reported by Wacker and Dorfman (1962) in urine of normal Americans.

Urinary LDH activity was measured for 60 Egyptian farmers infested with urinary schistosomiasis. Sixty per cent had normal LDH activity. Twenty per cent had high levels of LDH explained by marked haematuria and/or pyuria. Twenty per cent had high levels of LDH activity and were proved by clinicopathological or cytological methods to have bladder cancer complicating schistosomiasis.

The test is thus recommended to be carried out in schistosomal lesions of the urinary bladder whenever malignancy is suspected.

The authors are indebted to Captain L. F. Miller and Dr. F. O. Raasch, Jr., for supervising the study and reviewing the manuscript.

The opinions and assertions contained herein are the private ones of the authors and are not to be construed as official or reflecting the views of Ein Shams University Hospital, the Navy Department or the naval service at large.

### REFERENCES

Amador, E., Zimmerman, T. S. and Wacker, W. E. C.—(1963) *J. Am. med. Ass.*, **185**, 769.

Dubach, V. C. and Rediger, R.—(1964) *Urol. int.*, **17**, 65.

El Sebai, I.—(1962) 'Cancer of the Bladder in Egypt, U.A.R.' *Proc. int. Symp. on Bilharziasis*. Cairo. Part I, p. 709.

Kark, R. M., Lawrence, J. R., Pollak, V. E., Pirani, C. L., Muehrcke, R. C. and Silva, H.—(1963) 'A Primer of Urinalysis'. 2nd edition. New York (Hoeber Medical Division).

Papanicolaou, G. N.—(1954) 'Atlas of Exfoliative Cytology'. Cambridge, Mass. (Harvard University Press).

Wacker, W. E. C. and Dorfman, L. E.—(1962) *J. Am. med. Ass.*, **181**, 972.

Wacker, W. E. C., Dorfman, L. E. and Amador, E.—(1964) *J. Am. med. Ass.*, **188**, 671.